# Altered serum levels of kynurenine metabolites in patients affected by cluster headache

**DOI:** 10.1186/s10194-016-0620-2

**Published:** 2016-03-22

**Authors:** Martina Curto, Luana Lionetto, Andrea Negro, Matilde Capi, Francesca Perugino, Francesco Fazio, Maria Adele Giamberardino, Maurizio Simmaco, Ferdinando Nicoletti, Paolo Martelletti

**Affiliations:** Department of Psychiatry, Harvard Medical School, Boston, MA USA; Department of Molecular Medicine, Sant’Andrea Medical Center, Sapienza University of Rome, Via di Grottarossa 1035-1039, Rome, 00189 Italy; Advanced Molecular Diagnostics, IDI-IRCCS, Rome, Italy; Regional referral headache center, Sant’Andrea Hospital, Rome, Italy; IRCCS Neuromed, Pozzilli, Italy; Headache Center and Geriatrics Clinic, Department of Medicine and Science of Aging, “G. D’Annunzio” University, Chieti, Italy; Department of Physiology and Pharmacology, Sapienza University of Rome, Rome, Italy

**Keywords:** Kynurenine, Cluster headache, Glutamate, Pain, NMDA receptors

## Abstract

**Background:**

The reported efficacy of memantine in the treatment of patients with cluster headache (CH) suggests that NMDA receptors are involved in mechanisms of nociceptive sensitization within the trigeminal system associated with CH. NMDA receptors are activated or inhibited by neuroactive compounds generated by tryptophan metabolism through the kynurenine pathway. In the accompanying manuscript, we have found that serum levels of all kynurenine metabolites are altered in patients with chronic migraine. Here, we have extended the study to patients affected by episodic or chronic CH as compared to healthy controls.

**Method:**

We assessed serum levels of kynurenine (KYN), kynurenic Acid (KYNA), anthranilic acid (ANA), 3-hydroxy-anthranilic acid (3-HANA), 3-hydroxykynurenine (3-HK), xanthurenic acid (XA), quinolinic acid (QUINA), tryptophan (Trp) and 5-hydroxyindolacetic acid (5-HIAA) by means of a liquid chromatography/tandem mass spectrometry (LC/MS-MS) method in 21 patients affected by CH (15 with episodic and 6 with chronic CH), and 35 age-matched healthy subjects. Patients with psychiatric co-morbidities, systemic inflammatory, endocrine or neurological disorders, and mental retardation were excluded.

**Results:**

LC/MS-MS analysis of kynurenine metabolites showed significant reductions in the levels of KYN (-36 %), KYNA (-34 %), 3-HK (-51 %), 3-HANA (-54 %), XA (-25 %), 5-HIAA (-39 %) and QUINA (-43 %) in the serum of the overall population of patients affected by CH, as compared to healthy controls. Serum levels of Trp and ANA were instead significantly increased in CH patients (+18 % and +54 %, respectively). There was no difference in levels of any metabolite between patients affected by episodic and chronic CH, with the exception of KYN levels, which were higher in patients with chronic CH.

**Conclusion:**

The reduced levels of KYNA (an NMDA receptor antagonist) support the hypothesis that NMDA receptors are overactive in CH. A similar reduction in KYNA levels was shown in the accompanying manuscript in patients affected by chronic migraine. The reduced levels of XA, a putative analgesic compound, may contribute to explain the severity of pain attacks in CH. These data, associated with the data reported in the accompanying manuscript, supports a role for the kynurenine pathway in the pathophysiology of chronic headache disorders.

## Background

Cluster headache (CH), the most frequent primary headache disorder among the trigeminal autonomic cephalalgias, can be chronic or episodic depending on the presence of remission periods between the headache bouts [1–5]. The molecular events underlying the pathophysiology of CH are unknown because of the lack of valuable experimental animal model, and, therefore the treatment is largely based on empirical evidence [6]. It is intuitive that the hypothalamus and the trigeminal system are involved in the pathophysiology of CH. The involvement of the hypothalamus is suggested by the circannual and circadian periodicity of headache and by the prevalence of CH in males. In addition, changes in the secretion of hormones regulated by the hypothalamus, such as prolactin, growth hormone and cortisol are found in patients affected by CH [7–9], and functional imaging studies showed the activation of hypothalamic gray matter during headache attacks [6, 10]. Headache pain of CH is conveyed to the CNS *via* the ophthalmic branch of the trigeminal system, and, similarly to migraine, activation of the trigeminovascular system has been demonstrated during CH attacks [11]. Mechanisms of nociceptive sensitization developing at the synapses between primary afferent fibers and secondary order neurons of the caudal trigeminal nucleus, and in upper regions of the pain neuraxis likely contribute to the development of the typical unilateral attacks of pain associated with CH. Glutamate acting at N-methyl-D-aspartate (NMDA) receptors plays a key role in the induction of nociceptive sensitization [12], and this suggests that alterations in NMDA receptor signaling or in the endogenous machinery that activates NMDA receptors may be relevant to the pathophysiology of CH. It is consistent with this hypothesis that memantine, a fast off-rate NMDA-gated ion channel blocker, has shown efficacy in reducing CH attacks in resistant patients, even if clinical studies are still limited [13].

The kynurenine pathway of tryptophan metabolism generates neuroactive metabolites that influence the activity of NMDA receptors as well as other glutamate receptor types [14, 15]. In this pathway, L-tryptophan is first metabolized into N-formyl-kynurenine, which is then converted into L-kynurenine (KYN). KYN is then transaminated into KYNA by kynurenine aminotransferases (KATs), or, alternatively, converted into 3-hydroxykynurenine (3-HK) by kynurenine monoxygenase or transformed into anthranilic acid (ANA) by kynureninase. 3-HK is the precursor of 3-hydroxyanthranilic acid (3-HANA), quinolinic acid (QUINA), and xanthurenic acid (XA) (reviwed by Schwarcz et al. 2012) [16]. QUINA is an NMDA receptor agonist, whereas KYNA blocks the action of the co-agonist, glycine, at the GluN1 subunit of NMDA receptors (reviewed by Schwarcz et al. 2012) [16]. XA activates mGlu2 and mGlu3 metabotropic glutamate receptors, although its precise mechanism of action is unknown [17]. In the accompanying manuscript we showed for the first time that chronic migraine is associated with alterations in serum levels of kynurenine metabolites, which are in line with the hypothesis of a hyperactivity of NMDA receptors in migraine. Since hyperactive NMDA receptors play a role in nociceptive sensitization, we hypothesized that kynurenine glutamatergic metabolites might be altered in CH and extended the analysis to patients affected by episodic or chronic CH as compared to age-matched healthy controls.

## Methods

### Patients

The protocol was carried out in accordance with the declaration of Helsinki and the study design was reviewed and approved by the Ethical Committee at Sapienza, University of Rome, Sant’Andrea Hospital. All subjects signed free informed consent for participation in the study. All subjects were enrolled by the Regional Referral Headache Center of S. Andrea Hospital and evaluated by two experts in headache disorders (A.N. and P.M.). 21 patients met the ICHD-3beta criteria [1] for cluster headache, chronic or episodic, and were included in the CH group and 35 healthy volunteers, recruited among the Hospital and University employees, were included in the age-matched control group. Inclusion criteria for CH patients were: (i) age between 18 and 65 years; (ii) patients affected by CH during the active phase (ICHD-3beta criteria) [1]; (iii) patients treated with verapamil (120–480 mg) for prophylactic therapy and sumatriptan (oral 50 mg, subcutaneous 6 mg) as the acute pain medication.

Exclusion criteria for both patients and controls were: (i) the presence of psychiatric co-morbidities, systemic inflammatory disorders, endocrine disorders, neurological disorders, and mental retardation; (ii) lifetime history of cluster headache (for healthy volunteers); and (iii) the use of any drug of abuse in the last 3 months (except cigarette smoking).

A detailed medical anamnesis was obtained from patients and controls, and the daily frequency of cluster headache attacks was recorded for each patient. Patients were sampled during the CH active phase, independently of the presence of an ongoing attack. Sumatriptan intake was allowed until 12 h before the blood sample.

### Blood collection and KP measurement

Serum samples from patients and controls with empty stomach were collected between 10 am and 12 am. Blood (5 ml) was sampled in anticoagulant-free tubes and kept at room temperature for 1 h before the serum was isolated (centrifugation at 2000 g for 10 min at 20 °C). Aliquots of serum were stored at -80 °C until analysis.

We developed a reliable liquid chromatography/tandem mass spectrometry (LC/MS-MS) method for the assay of serum levels of all kynurenine metabolites. The method allowed a reliable detection of KYN, KYNA, ANA, 3-HANA, 3- HK, XA, and QUINA. Levels of tryptophan (Trp) and 5-hydroxyindolacetic acid (5-HIAA) were also detected. Details on sample preparation, reagents, standard solutions, chromatographic conditions, mass spectrometry conditions, and validation parameters are reported in Tables S1-S3 of Fazio et al. 2015 [17].

### Statistical analysis

SPSS version 19.00 (IBM Corporation, Armonk NY, USA) was used for data analysis. Continuous variables were expressed as mean standard ± deviation (S.D.), while discrete variables were assigned as numbers and percentages. The compliance of continuous variables with normal distribution was controlled with the Kolmogorov–Smirnov test. Whether Healthy Controls and Cluster Headache groups differed in terms of discrete variables was checked through Pearson’s Chi Squared test (χ2). Since continuous variables did not comply with a normal distribution, the Mann–Whitney U test was used for between group comparisons. Spearman’s Rho correlation (ρ) was used to test between-variables relationships. We set statistical significance at *P* ≤ 0.05.

## Results

### Patients characteristics and serum levels of kynurenine metabolites

Demographic and clinical features of patients affected by CH (*n* = 21) and age-matched healthy controls (*n* = 35) are shown in Table 1. The CH group, as expected, showed a prevalence of male gender (90.5 %), which was not significantly higher than in the group of selected HCs (77.1 %). Mean age was similar between the two groups (about 44 years) and mean frequency of headache attacks in CH patients was about 2 attacks/day. Six patients were affected by chronic CH, and the remaining 15 patients were affected by episodic CH. The latter patients were examined during the active phase of CH.

**Table 1:** Demographic and clinical characteristics and serum kynurenine pathways metabolites levels in the study groups

	Healthy controls (*n* = 35)	Patients with CH (*n* = 21)	χ^2^/U *p*
Gender (M, %)	27 (77.1)	19 (90.5)	1.59
			0.19
Age (years; mean ± SD)	44.8 ± 7.64	44,4 ± 7.60	278
			0.13
Chronic cluster headache (n, %)	--	6 (28.6 %)	--
Mean daily frequency of attacks (mean ± SD)	--	2.05 ± 0.86	--
Trp (μg/ml) (mean ± SD)	5.50 ± 1.71	6.50 ± 1.60	233
			*0.01*
KYN (μg/ml) (mean ± SD)	0.36 ± 0.15	0.23 ± 0.11	165.5
			*<0.01*
KYNA (ng/ml) (mean ± SD)	3.15 ± 1.39	2.09 ± 1.49	189
			*0.03*
ANA (ng/ml) (mean ± SD)	1.85 ± 1.57	2.85 ± 1.37	199
			*<0.01*
3-HK (ng/ml) (mean ± SD)	2.16 ± 1.58	1.05 ± 0.84	217
			*0.01*
3-HANA (ng/ml) (mean ± SD)	9.76 ± 4.38	4.50 ± 1.52	67.5
			*<0. 01*
QUINA (ng/ml) (mean ± SD)	16.3 ± 10.1	9.28 ± 2.28	115
			*<0.01*
5-HIAA (ng/ml) (mean ± SD)	33.4 ± 13.7	20.5 ± 8.36	156.5
			*<0.01*
XA (ng/ml) (mean ± SD)	1.77 ± 0.95	1.32 ± 1.53	148
			*0.02*

**Fig. 1:**
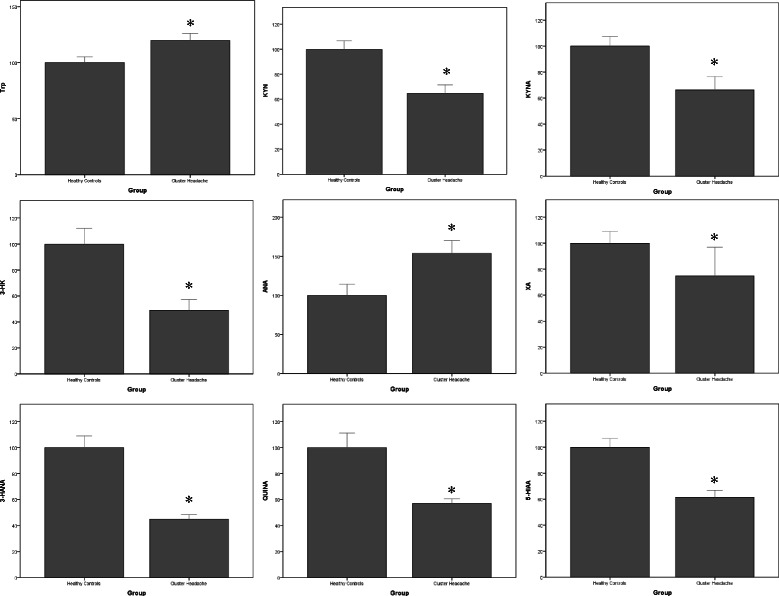
Percent changes in serum kynurenine metabolites in patients affected by CH with respect to healthy controls. Data are presented as per cent of HC values for each metabolite. Values are means ± S.E.M. *p < 0.05 vs. healthy controls (HCs)

LC/MS-MS analysis of kynurenine metabolites showed significant reductions in serum levels of KYN (-36 %), KYNA (-34 %), 3-HK (-51 %), 3-HANA (-54 %), XA (-25 %), 5-HIAA (-39 %) and QUINA (-43 %) in the serum of the overall population of patients affected by CH, as compared to HCs (Table 1, Fig. 1). Serum levels of Trp and ANA were instead significantly increased in patients affected by CH (+18 % and +54 %, respectively).

### Kynurenine metabolites levels in the serum of patients with chronic and episodic CH, and correlations with daily headache frequency

Analysis of serum levels of kynurenine metabolites in patients affected by episodic or chronic CH showed no differences between the two subgroups, with exception of KYN levels, which were significantly higher (+50 %) in patients affected by chronic CH (Table 2).

**Table 2:** Kynurenine pathway metabolites in Chronic and Episodic Cluster Headache groups

	Episodic CH (*n* = 15)	Chronic CH (*n* = 6)	U *p*
Trp (μg/ml) (mean ± SD)	6.60 ± 1.74	6.57 ± 1.32	43.5
			0.91
KYN (μg/ml) (mean ± SD)	0.20 ± 0.07	0.31 ± 0.16	20.0
			*0.05*
KYNA (ng/ml) (mean ± SD)	1.91 ± 1.43	2.52 ± 1.67	34.0
			0.42
ANA (ng/ml) (mean ± SD)	2.67 ± 1.44	3.30 ± 1.18	30.0
			0.27
3-HK (ng/ml) (mean ± SD)	1.21 ± 0.94	0.66 ± 0.29	30.5
			0.27
3-HANA (ng/ml) (mean ± SD)	4.44 ± 1.69	4.22 ± 1.11	42.0
			0.85
QUINA (ng/ml) (mean ± SD)	9.33 ± 2.36	8.54 ± 2.17	7.00
			0.93
5-HIAA (ng/ml) (mean ± SD)	18.4 ± 6.32	25.8 ± 10.9	21.0
			0.07
XA (ng/ml) (mean ± SD)	1.33 ± 1.59	1.18 ± 1.07	6.00
			0.93

No significant correlations were found between any kynurenine metabolite and daily frequency of headache attacks (not shown).

## Discussion

The profile of serum kynurenine metabolites in patients affected by CH was similar to that reported in the accompanying manuscript in patients with chronic migraine with two notable exceptions: (i) XA levels, which were decreased in patients with CH, but increased in patients with chronic migraine; and, (ii) ANA levels, which were increased to a much lower extent in patients with CH than in patients with chronic migraine. As we have highlighted in the accompanying manuscript, serum levels of kynurenine metabolites may roughly mirror CNS levels because the constitutive action of the kynurenine pathway is low in the CNS, and at least KYN and 3-HK of peripheral source can cross the blood-brain barrier and fuel the pathway in the brain parenchyma [16]. The most relevant finding here is the reduction in KYNA levels, which, within the context of an enhanced release of glutamate might contribute to a hyperactivity of NMDA receptors resulting into nociceptive sensitization in CH. Thus, the reductions in KYNA levels found in patients affected by CH (present data) and in patients affected by chronic migraine (see accompanying manuscript) represent a nice clinical counterpart of the numerous experimental data that demonstrate the ability of KYNA to restrain the activation of the trigeminal system [18–22]. In fact, several preclinical studies demonstrated the mediatory and modulatory role of KYNA for glutamate and its receptors in central and peripheral pain processing [23]. Some of the identified roles are: the attenuation of the cutaneous nociceptors sensitization by the inactivation of peripheral glutamate receptors [24], the probable reduction of the first-order neurons activation and of the consecutive release of CGRP and nitric oxide from the nerve endings in the trigeminal nucleus caudalis [22] and the inhibition of the neural activity on brain stem structures such as the locus coeruleus, periaqueductal grey and nucleus raphe magnus [25–28].

The different behavior of XA in patients by CH and chronic migraine is interesting and deserves some comments. XA is a putative neurotransmitter [29] that activates mGlu2 and mGlu3 metabotropic glutamate receptors [17], and may also indirectly influence glutamatergic transmission by interacting with vesicular glutamate transporters [30]. Activation of mGlu2 receptors causes analgesia by negatively modulating glutamate release from primary afferent fibers [31], and preliminary data demonstrate the analgesic activity of XA in rodents (M. Bernabucci and F. Fazio, unpublished observation). Hence, the increase in serum XA levels found in patients with chronic migraine has been interpreted as a defensive mechanism aimed at reducing the extent of headache in migraine. This potential defense was absent in patients with CH, where serum XA levels were reduced. We speculate on the possibility that a reduced production of XA contributes to the severity of pain attacks in CH, and that pharmacological strategies aimed at enhancing XA biosynthesis or reducing XA catabolism might cause analgesia in CH patients. This attractive hypothesis warrants further investigation. ANA levels were found to be increased in patients affected by both CH and chronic migraine, suggesting that, in both disorders, KYN is preferentially metabolized by kynureninase rather than by kynurenine monoxygenase or kynurenine aminotransferases (see also accompanying manuscript). Why ANA levels were much more elevated in chronic migraine than in CH is unknown. The only biochemical explanation is that ANA transformation into 3-HANA or other catabolites is dramatically reduced in chronic migraine, but not in CH. The incomplete knowledge of the metabolism of ANA makes this hypothesis difficult to prove. The marked difference in ANA levels between chronic migraine and CH suggests that this compound may serve as a peripheral biomarker that may facilitate the correct diagnosis of patients with chronic headache with mixed clinical features.

Alterations of serum kynurenine pathway metabolites have been found in other neuropsychiatric disorders [16], have been linked to chronic stress [32] and sleep deprivation [33]. Although we did not recruit patients affected by psychiatric comorbidities, we cannot exclude that the chronic stress associated to the active CH condition, often associated with sleep disturbances, might have affected tryptophan metabolism.

## Conclusion

In conclusion, we have shown for the first time that CH is associated with abnormalities of the kynurenine pathway of tryptophan metabolism, as reflected by substantial alterations of all kynurenine metabolites in the peripheral blood. This manuscript and the accompanying manuscript reporting data obtained in patients with chronic migraine are in line with the increasing number of preclinical data that highlight the importance of the kynurenine pathway in the pathophysiology and treatment of chronic headache disorders.
